# CT‐based whole lung radiomics nomogram to identify middle‐aged and elderly COVID‐19 patients at high risk of progressing to critical disease

**DOI:** 10.1002/acm2.14562

**Published:** 2024-11-29

**Authors:** Xin'ang Jiang, Jun Hu, Qinling Jiang, Taohu Zhou, Fei Yao, Yi Sun, Chao Zhou, Qianyun Ma, Jingyi Zhao, Kang Shi, Wen Yang, Xiuxiu Zhou, Yun Wang, Shiyuan Liu, Xiaoyan Xin, Li Fan

**Affiliations:** ^1^ Department of Radiology Second Affiliated Hospital of Naval Medical University Shanghai China; ^2^ Department of Radiology Nanjing Drum Tower Hospital The Affiliated Hospital of Nanjing University Medical School Nanjing Jiangsu China; ^3^ School of Medical Imaging Weifang Medical University Weifang Shandong China; ^4^ School of Medicine Shanghai University Shanghai China

**Keywords:** COVID‐19, elderly, middle‐aged, nomogram, pneumonia, tomography, X‐ray computed

## Abstract

**Background:**

COVID‐19 remains widespread and poses a threat to people's physical and mental health, especially middle‐aged and elderly individuals. Early identification of COVID‐19 patients at high risk of progressing to critical disease helps improve overall patient outcomes and healthcare efficiency.

**Purpose:**

To develop a radiomics nomogram to predict the risk of newly admitted middle‐aged and elderly COVID‐19 patients progressing to critical disease.

**Methods:**

A total of 794 patients (aged 40 years or above) were retrospectively included in the study from two institutions, all of them were with non‐critical COVID‐19 on admission. At follow‐up, patients were divided into non‐critical group and critical group. About 443 patients (384 non‐critical and 59 critical) from the first hospital were randomly assigned to the training (*n* = 311) and internal validation (*n* = 132) set in a 7:3 ratio. Additionally, an independent external cohort of 351 patients (292 non‐critical and 59 critical) from another hospital was evaluated. Radiomics signatures and clinical indicators were used to build a radiomics model and a clinical model after computed tomography (CT) image processing, CT whole‐lung segmentation, feature extraction, and feature selection. The radiomics nomogram model integrated radiomics model and clinical model. The receiver operating characteristic curve (AUC) was used to assess the performance of the proposed models. Calibration curves and decision curve analysis were used to assess the performance of the radiomics nomogram.

**Results:**

For the training, internal validation, and external validation sets, the AUC values of the radiomic nomogram for the prediction of COVID‐19 progression were 0.916, 0.917, and 0.890, respectively. Calibration curves indicated that there was no significant departure between prediction and observation in three sets. The decision curve image demonstrated the clinical utility of the nomogram model.

**Conclusions:**

Our nomogram model incorporates radiomics features and clinical indicators, it provides a new pathway to increase predictive accuracy or clinical utility, further helping to provide personalized management for middle‐aged and elderly patients with COVID‐19.

## 1 BACKGROUND

Coronavirus disease 2019 (COVID‐19), was initially identified in China in 2019 and subsequently spread worldwide, exploding into a pandemic. In May 2023, the World Health Organization declared that COVID‐19 pandemic no longer constitutes a public health emergency of international concern (PHEIC). However, COVID‐19 remains widespread and poses a threat to people's physical and mental health, with a wide range of clinical manifestations ranging from asymptomatic or mild respiratory symptoms to severe pneumonia and acute respiratory distress syndrome (ARDS). Notably, middle‐aged and elderly individuals have been identified as a high‐risk population for the development of severe or critical COVID‐19 pneumonia, leading to increased morbidity and mortality rates within this demographic group.^1^, ^2^ Early identification of COVID‐19 patients at high risk of progressing to critical disease is crucial for prompt clinical intervention and resource allocation, thereby improving overall patient outcomes and healthcare efficiency. Laboratory parameters were shown to help identify high‐risk patients in many COVID‐19 studies.^3^, ^4^, ^5^ However, laboratory tests still have limitations such as poor stability, lag time and invasiveness.

Recent advancements in medical imaging, particularly computed tomography (CT), have made the diagnosis and complication detection of COVID‐19 patients more accurate and efficient.^6^ Chest CT is widely used in the diagnosis and the assessment of treatment effectiveness of COVID‐19 because of its intuitive presentation and fast scanning time. Nonetheless, difficulties still remain in predicting the progression of COVID‐19 to critical disease by relying solely on a single CT examination. Radiomics, a rapidly evolving field within medical imaging, has been widely used in lung diseases such as pulmonary nodules, chronic obstructive pulmonary disease, pneumonia, and so on,^7^, ^8^, ^9^ facilitating a deeper characterization of disease heterogeneity. With the abundance of quantitative information provided by CT images, radiomics analysis can offer “imaging biomarker” that aid in the early identification of COVID‐19 patients at a higher risk of developing critical COVID‐19 pneumonia.

To the best of our knowledge, no radiomics studies focusing on middle‐aged and elderly patients with COVID‐19 have been reported. However, cohort within this age range, recognized as a high‐risk population, should be the focus of increased attention. Alveolar fibrinous exudate with hyaline membranes, reactive pneumocytes, and exudative widespread alveolar damage with alveolar and interstitial edema are the pathological features of COVID‐19 pneumonia.^10^ We believe that exudative alveolar pneumonia may induce alterations across both lungs. However, previous radiomics studies on COVID‐19 pneumonia have predominantly concentrated on pneumonia segments presenting as high‐density opacities on CT images, rather than employing a holistic radiomic approach covering the whole lung.^11^, ^12^, ^13^ In our research, we focused on the whole lung by using a deep learning model to segment lung tissue from chest CT scans, this deep learning model automates the segmentation process, ensuring accurate and consistent delineation of lung structures. Using radiomics techniques, we attempted to uncover the underlying information to provide a more comprehensive understanding of the prognosis or outcome of COVID‐19 patients.

Therefore, this study aimed to develop a CT‐based whole lung radiomics nomogram for predicting the risk of newly admitted middle‐aged and elderly COVID‐19 patients progressing to critical disease.

## 2 METHODS

### 2.1 Study participants and groups

**FIGURE 1:**
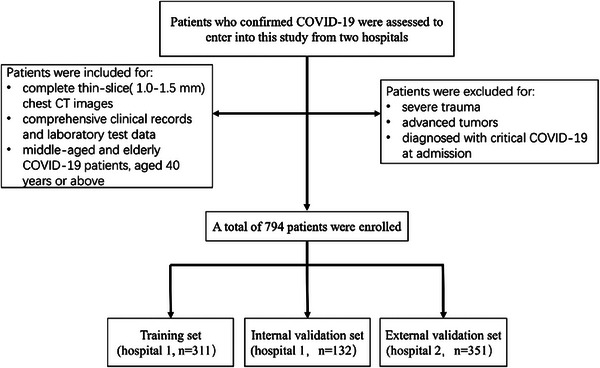
The flowchart of the inclusion and exclusion criteria.

This study evaluated all patients diagnosed with COVID‐19 who presented at Second Affiliated Hospital of Naval Medical University (Hospital 1) and Nanjing Drum Tower Hospital (Hospital 2) during the period from December 17 2022 to January 31 2023. All of the patients had COVID‐19 confirmed by positive reverse transcriptase–polymerase chain reaction (RT‐PCR) tests for SARS‐COV‐2 from throat‐swab specimens.^14^ The following criteria were applied for the inclusion of study participants: (1) complete thin‐slice(1.0–1.5 mm) chest CT images; (2) comprehensive clinical records and laboratory test data; (3) middle age (people aged 40–59 years) and elderly (people aged 60 years or older). Exclusion criteria included severe trauma or advanced tumors, they are the clinical confounding factors that may have effects on the prognosis of COVID‐19 patients. Patients diagnosed with critical COVID‐19 at admission were also excluded because the purpose of our study is to predict the progression of COVID‐19 to critical disease (Figure 1). According to the “Diagnosis and treatment protocol for COVID‐19 patients (Trial Version 9)” recommended by China's National Health Commission, Participants meeting any of the following criteria were included in the critical group: (1) respiratory failure occurred and mechanical ventilation required; (2) shock; (3) other organ failure needing intensive care unit (ICU) monitoring treatment.^15^ Furthermore, patients with a fatal outcome were also included in the critical group. Finally, patients were classified into two cohorts: the critical group and the non‐critical group. All patients were followed up until 1 month after the diagnosis of COVID‐19, hospital discharge, or death by electronic medical record system or telephone follow‐up.

At follow up, a total of 794 patients (443 from Hospital 1 and 351 from Hospital 2) were enrolled in our study. Each of the two hospitals had 59 patients included in the critical group. Then, 443 patients from Hospital 1 were randomized into the training (*n* = 311) or internal validation dataset (*n* = 132) in a 7:3 ratio using the R package (version 4.3.1 https://www.R‐project.org). A total of 351 patients from Hospital 2 served as an independent external validation. Features for model training are only from the training set.

### 2.2 CT image acquisition and clinical data collection

Since this is a dual‐center study, all non‐enhanced chest CT images were acquired using multi‐slice CT systems from four different manufacturers, United‐imaging, Philips, SIEMENS and GE (detailed scan and reconstruction parameters are shown in Appendix 1). Thin‐section (1.0–1.5 mm) chest CT images [window width, 1500 Hounsfield units (HU); window level−600 HU] were used for further radiomics analysis.

The clinical characteristics including demographics, underlying diseases (hypertension, coronary heart disease, chronic lung disease, diabetes, chronic liver disease and chronic kidney disease), clinical symptoms and laboratory test data were extracted from electronic medical records (Table 1). Laboratory tests included arterial blood gas analysis and routine blood tests. Arterial blood gas analysis included lactate, PaO_2_, PaCO_2_, and pH. Considering arterial blood is collected under different oxygen‐inhaling states of the patient, some patients didn't even receive oxygen supplementation, PaO_2_ and PaCO_2_ and pH were not used in the clinic's model because we believe that they may not reflect the patient's actual baseline status.

**TABLE 1:** Characteristics of patients for clinical model construction.

	Training set (*n* = 311, hospital 1)			Internal validation set (*n* = 132, hospital 1)			External validation set (*n* = 351, hospital 2)		
Characteristic	Non‐critical (*n* = 263)	Critical (*n* = 48)	*p*	Non‐critical (*n* = 121)	Critical (*n* = 11)	*p*	Non‐critical (*n* = 292)	Critical (*n* = 59)	*p*
Gender, *n* (%)			0.147			0.062			0.762
Male	163 (61.98)	35 (72.92)		74 (61.16)	3 (27.27)		192 (65.75)	40 (67.80)	
Female	100 (38.02)	13 (27.08)		47 (38.84)	8 (72.73)		100 (34.25)	19 (32.20)	
Age, mean ± SD	70.81 ± 14.29	77.23 ± 11.95	0.004	70.46 ± 14.85	70.64 ± 12.44	0.970	65.93 ± 14.59	71.85 ± 16.16	0.006
Smoke, *n* (%)			0.771			1.000			0.121
No	240 (91.25)	45 (93.75)		111 (91.74)	10 (90.91)		255 (87.33)	47 (79.66)	
Yes	23 (8.75)	3 (6.25)		10 (8.26)	1 (9.09)		37 (12.67)	12 (20.34)	
Comorbidy, *n* (%)									
Hypertension	155 (58.94)	30 (62.50)	0.644	71 (58.68)	5 (45.45)	0.595	147 (50.34)	34 (57.63)	0.307
Coronary heart disease	57 (21.67)	12 (25.00)	0.610	21 (17.36)	3 (27.27)	0.683	41 (14.04)	17 (28.81)	0.005
Chronic lung disease	18 (6.84)	7 (14.58)	0.127	13 (10.74	1 (9.09)	1.000	30 (10.27)	10 (16.95)	0.217
Diabetes	82 (31.18)	15 (31.25)	0.992	46 (38.02)	4 (36.36)	1.000	72 (24.66)	18 (30.51)	0.348
Chronic liver disease	18 (6.84)	5 (10.42)	0.569	13 (10.74)	1 (9.09)	1.000	10 (3.42)	5 (8.47)	0.163
Chronic kidney disease	50 (19.01)	17 (35.42)	0.011	25 (20.66)	2 (18.18)	1.000	28 (9.59)	17 (28.81)	<0.001
Symptoms, *n* (%)									
Cough	200 (76.05)	37 (77.08)	0.877	92 (76.03)	6 (54.55)	0.230	171 (58.56)	37 (62.71)	0.554
Fever	186 (70.72)	36 (75.00)	0.547	86 (71.07)	5 (45.45)	0.156	257 (88.01)	54 (91.53)	0.439
Sore throat	16 (6.08)	2 (4.17)	0.852	5 (4.13)	0 (0.00)	1.000	54 (18.49)	8 (13.56)	0.365
Muscle soreness	24 (9.13)	4 (8.33)	0.161	11 (9.09)	0 (0.00)	0.598	32 (10.96)	7 (11.86)	0.840
SaO_2_ (%)	96.02 ± 2.65	92.54 ± 3.32	<0.001	96.20 ± 1.50	91.76 ± 2.87	<.001	95.86 ± 2.18	89.72 ± 6.67	<0.001
pH	7.42 ± 0.03	7.43 ± 0.04	0.139	7.42 ± 0.04	7.43 ± 0.01	0.688	7.44 ± 0.04	7.43 ± 0.09	0.896
PaCO_2_ (mmHg)	36.51 ± 3.74	33.53 ± 6.10	0.002	36.16 ± 3.46	37.14 ± 2.28	0.356	36.95 ± 4.26	37.03 ± 11.07	0.953
PaO_2_ (mmHg)	91.95 ± 14.87	76.91 ± 24.54	<0.001	92.60 ± 13.61	72.96 ± 12.56	<.001	91.41 ± 25.55	64.55 ± 22.23	<0.001
ALac (mmol/L)	1.45 ± 0.44	1.80 ± 0.63	<0.001	1.41 ± 0.32	2.01 ± 0.90	0.051	1.26 ± 0.33	2.07 ± 1.07	<0.001
WBC (×10^9^/L)	6.10 ± 2.90	9.81 ± 6.16	<0.001	6.19 ± 2.89	11.42 ± 7.37	0.041	6.51 ± 4.89	8.72 ± 8.36	0.053
Neutrophils (×10^9^/L)	4.55 ± 2.80	8.51 ± 5.86	<0.001	4.72 ± 2.71	10.01 ± 6.44	0.022	4.50 ± 3.85	5.93 ± 3.87	0.010
Lymphocytes (×10^9^/L)	1.02 ± 0.57	0.69 ± 0.36	<0.001	1.00 ± 0.63	0.95 ± 1.01	0.818	2.25 ± 18.73	0.95 ± 0.70	0.592
CRP (mg/L)	45.31 ± 42.33	87.85 ± 59.00	<0.001	43.58 ± 37.42	62.80 ± 66.58	0.133	50.98 ± 58.94	78.77 ± 77.01	0.011

Abbreviations: Alac, arterial lactate; CRP, C‐reactive protein; PaO_2_, partial pressure of oxygen in arterial blood; PaCO_2_, partial pressure of carbon dioxide in arterial blood; pH, arterial pH; SaO_2_, oxygen saturation; WBC, white blood cell.

In each case, the first CT scan and laboratory test data within 2 days after being diagnosed with COVID‐19 were collected.

### 2.3 Whole lung segmentation and radiomics features selection

Initially, we utilized a publicly available deep‐learning model called U‐net (R231) (https://github.com/JoHof/lungmask) to automatically segment the right and left lungs. This model was trained on extensive and diverse datasets, encompassing a wide range of visual variations. The extracted left and right lungs were combined into a unified region of interest (ROI). Subsequently, a thoracic radiologist with five years of experience independently evaluated the segmentation results using ITK‐SNAP software (version 3.8.0, www.itksnap.org) for accurate assessment and correction of any faulty segmentations. Before feature extraction, the image underwent a three‐step preprocessing procedure. These steps included voxel resampling, gray discretization, and image intensity normalization. Subsequently, Radiomics features were extracted using the open‐source software pyradiomics (version 3.0.1, https://pyradiomics.readthedocs.io/en/latest/). Three types of features were obtained: first‐order features, shape features, and texture features. Radiomics features of the entire lung, which exhibited considerable data dispersion, were processed by *z*‐score standardization. Finally, a total of 1218 radiomics features were extracted. Two feature selection methods, maximum relevance minimum redundancy (mRMR) and least absolute shrinkage and selection operator (LASSO), were used to select the feature. Before LASSO regression, mRMR was performed to eliminate the redundant and irrelevant features. Then LASSO was conducted to choose the optimized subset of features to construct the final model.

### 2.4 Model construction

The most significant radiomics features were selected to construct radiomics model based on logistic regression. Specifically, we applied the mRMR algorithm to assess the relevance and redundancy of the remaining features.^16^ The penalty parameter was adjusted by 10 cross‐validations. Some candidate features coefficients were shrunk to zero and the remaining variables with non‐zero coefficients were selected by LASSO regression. A rad‐score was calculated for each patient via a linear combination of selected features that were weighted by their respective coefficients and a logistic regression model based on rad‐score was constructed. Variables with *p* < 0.05 were included in a multivariable logistic regression analysis to select a combination of clinical factors and laboratory test data. Based on the selected clinical features, we established a clinical model to predict critical COVID‐19 pneumonia. Finally, we developed a combined model that integrates radiomics features with clinical features.

### 2.5 Model validation: internal and external

In the internal and external validation, we used data from 132 and 351 patients, respectively, to assess the model's performance. The data processing for two cohorts followed the same procedure as that for the model development cohort. To facilitate clinical use and validate the model's generalizability, we created a simple nomogram that combines clinical and radiomic features. Moreover, we employed the Hosmer‐Lemeshow (HL) test to construct a calibration curve, evaluating the consistency between the actual and predicted outcomes of COVID‐19 patients. Finally, we utilized Decision Curve Analysis (DCA) to discuss the potential clinical utility of our model.

### 2.6 Statistical analysis

The measurements were analyzed using SPSS software (version 25.0, IBM) and R software (version 4.3.1 https://www.R‐project.org). Mean ± standard deviation (SD) and proportions are used to express continuous and categorical variables, respectively. Shapiro–Wilk tests were used to determine the normality of quantitative variables. The independent‐sample *t* test was used to assess normally distributed data. The Mann–Whitney *U* test was used to assess nonnormally distributed data. For categorical variables, the chi‐square and Fisher exact tests were performed. *P* value < 0.05 was considered statistically significant. Receiver operating characteristic (ROC) curve was used to analyze and evaluate the prediction performance of the model. To determine whether the efficiency disparity between the models was statistically significant, the DeLong test was applied.

## 3 RESULTS

In this retrospective study, 794 patients (676 non‐critical cases and 118 critical cases) who were confirmed with COVID‐19 were included. The training set includes 311 patients (263 non‐critical cases [age 70.8 ± 14.3 years; male 61.2%] and 48 critical cases [age 77.2 ± 11.2 years; male 72.9%]). The internal validation set includes 132 patients (121 non‐critical cases [age 74.5 ± 14.9 years; male 61.2%] and 11 critical cases [age 70.6 ± 12.4 years; male 27.3%]). 351 (292 non‐critical cases [age 65.9 ± 14.6 years; male 65.8%] and 59 critical cases [age 71.9 ± 16.2 years; male 67.8%]) from Hospital 2 were included in an independent external validation set (Table 1).

### 3.1 Clinical data screening and model development

**FIGURE 2:**
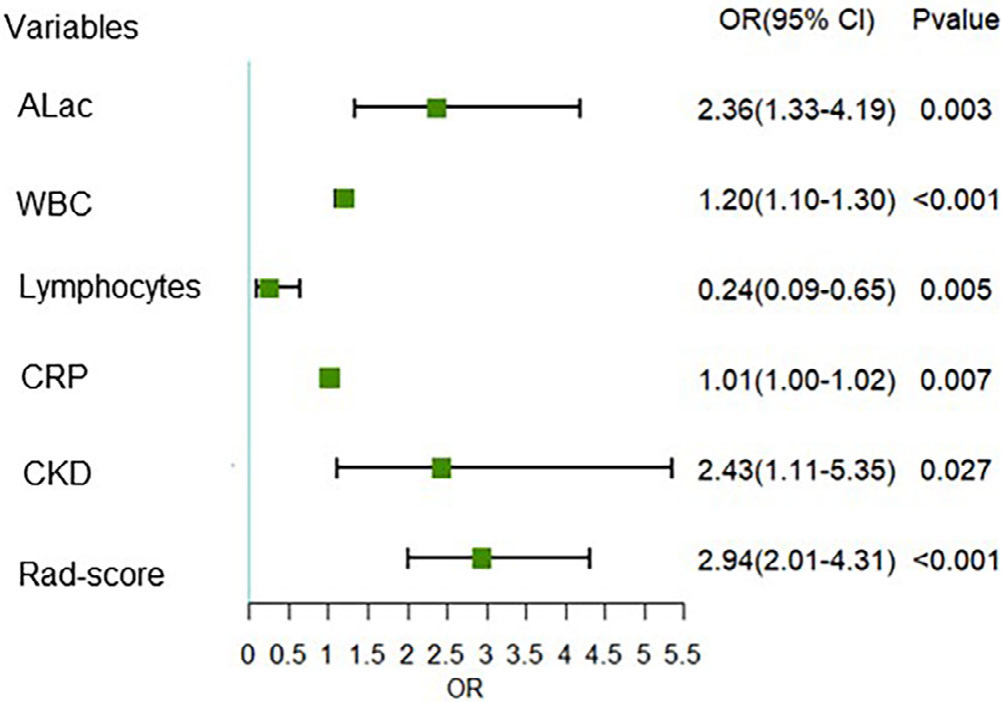
Multivariate analysis forest plot by logistic regression. ALac, arterial lactate; CKD, chronic kidney disease; CRP, C‐reactive protein; WBC, white blood cell.

Table 1 presents the detailed distribution of clinical characteristics in two groups. Gender and smoking history were found not significantly different between the two groups in all sets, while there was a significant difference in age between these two groups in the training set and external validation set. In the training set, patients in the critical group were more likely to have chronic kidney disease (CKD) than were those in the non‐critical group (17 [35.42%] vs. 50 [19.01%]; *p* = 0.011). Compared with those in the non‐critical group, patients in the critical group had worse laboratory test results, including SaO_2_, PaCO_2_, PaO_2_, arterial lactate (ALac), white blood cell (WBC), Neutrophils and Lymphocytes as well as C‐reactive protein (CRP) (*P* all <0.05). After univariable and multivariable logistic regression analyses, ALac, WBC, lymphocytes, CRP and CKD were selected as independent predictors to develop a clinical model. Results of univariate analysis are demonstrated in Appendix 2. To make the results more intuitive, a forest plot for the coefficients in the multivariable clinical model is shown in Figure 2. Finally, the clinical model obtained an AUC of 0.857 (95% confidence interval [CI]: 0.814–0.894) and accuracy of 80% (sensitivity, 77%; specificity, 81%) in the training set, an AUC of 0.850 (95% CI: 0.777–0.906) and accuracy of 73% (sensitivity, 100%; specificity, 70%) in the internal validation set and an AUC of 0.811(95% CI: 0.767–0.851) and accuracy of 70% (sensitivity, 86%; specificity, 66%) in the independent external validation set (Table 2).

**TABLE 2:** Performance of different models for predicting COVID‐19 patients progressing to critical disease.

Model		Training set (*n* = 311, hospital 1)				Internal validation set (*n* = 132, hospital 1)				External validation set (*n* = 351, hospital 2)			
	AUC (95%CI)	Cutoff	Sensitivity	Specificity	Accuracy	AUC (95%CI)	Sensitivity	Specificity	Accuracy	AUC (95%CI)	Sensitivity	Specificity	Accuracy
Clinical model	0.857 (0.814–0.894)	−1.69	0.77	0.81	0.80	0.850 (0.777–0.906)	1.00	0.70	0.73	0.811 (0.767–0.851)	0.86	0.66	0.70
Radiomics model	0.880 (0.839–0.914)	−1.08	0.77	0.87	0.85	0.859 (0.787–0.913)	1.00	0.62	0.65	0.839 (0.797–0.876)	0.86	0.71	0.73
Integrated nomogram model	0.916 (0.879–0.944)	−1.37	0.81	0.89	0.87	0.917 (0.857–0.958)	0.91	0.82	0.83	0.890 (0.852–0.921)	0.78	0.86	0.84

### 3.2 Feature selection and development of a radiomics signature

**FIGURE 3:**
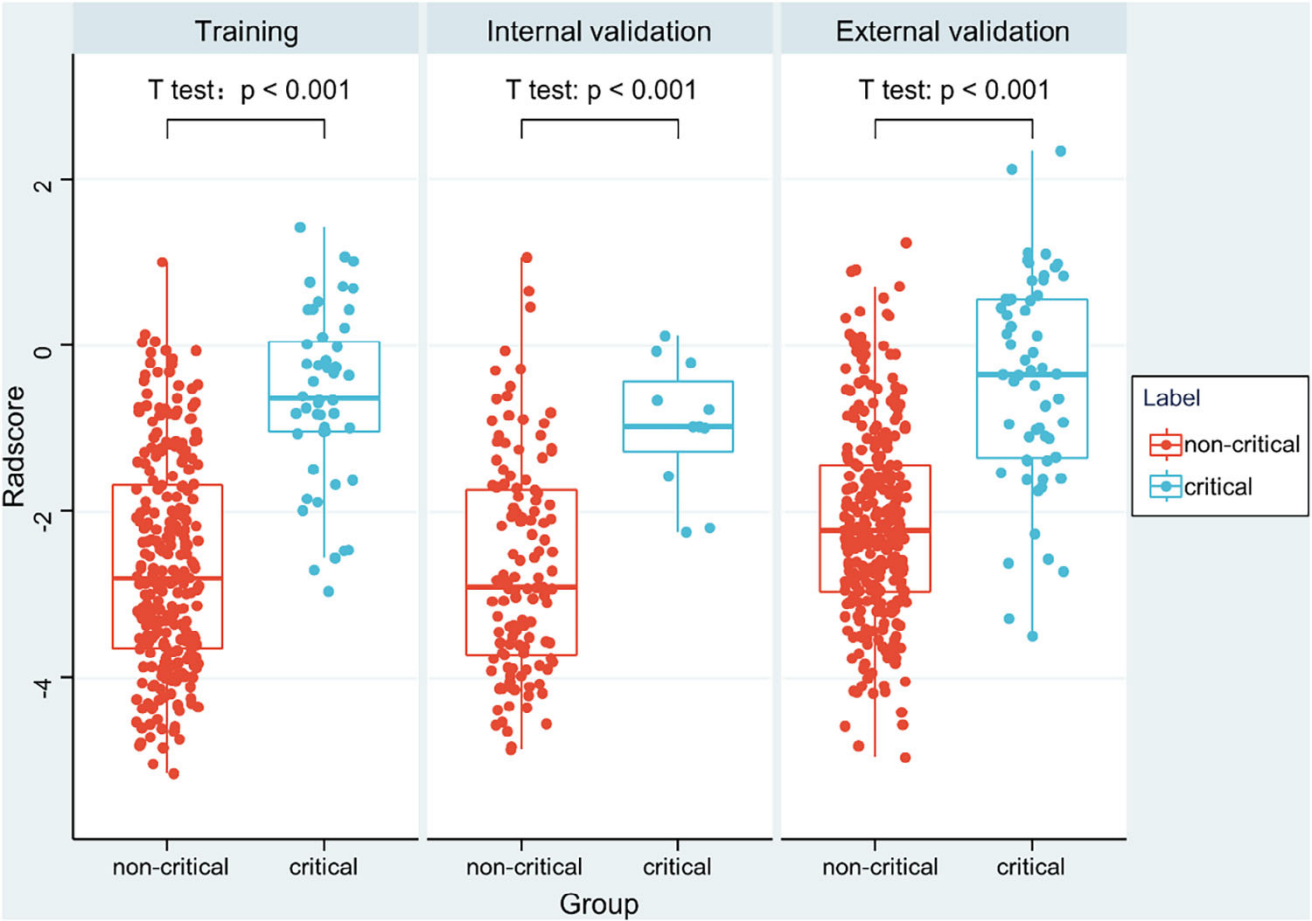
Box plot demonstrating the distribution of Radscores between two groups.

A total of 1218 radiomics features were extracted from CT images. At first, mRMR was performed to eliminate the redundant and irrelevant features, 20 features were retained. Then LASSO was conducted to choose the optimized subset of features to construct the final model. LASSO analysis included choosing the regular parameter *λ*, determining the number of the feature (Appendix 3a,b). Finally, the radiomics signature was constructed by using seven features (Appendix 3c), including two first‐order statistical features, two gray‐level size zone matrix features, two texture features, and one shape feature. The Radscores for each patient in all three sets are shown in Figure 3. Compared with those in the non‐critical group, patients in the critical group had a higher Radscore (*p* < 0.001). Univariate logistic regression analysis showed that patients with a higher Radscore may have a higher risk of suffering from critical illness (requiring ventilator support, shock, and even death) with the OR of 3.73 (95% CI: 2.58–5.38; *p* < 0.001) (Appendix 2). The radiomics model yielded AUCs of 0.880 (95% CI, 0.839–0.914), 0.859 (95% CI, 0.787–0.913) and 0.839 (95% CI, 0.797–0.876) in the training, internal validation and external validation sets, respectively.

The Radscore was calculated using the following formula: Radscore = 0.776*wavelet.LLL_gldm_DependenceEntropy+‐0.231*log.sigma.4.0.mm.3D_firstorder_RootMeanSquared+0.085*log.sigma.1.0.mm.3D_firstorder_Skewness+‐0.262*log.sigma.3.0.mm.3D_glszm_ZonePercentage+0.052*log.sigma.5.0.mm.3D_glszm_LowGrayLevelZoneEmphasis+0.275*original_shape_Elongation+‐0.269*wavelet.LHL_glcm_Idmn + ‐2.331.

### 3.3 Construction of the radiomics nomogram and performance comparison

**FIGURE 4:**
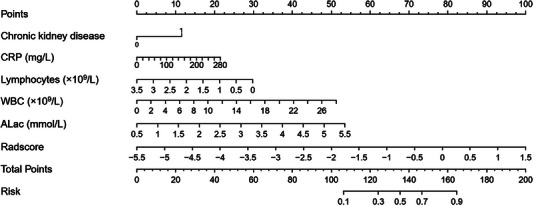
Developed integrated nomogram model. The nomogram is constructed by combining ALac, WBC, Lymphocytes, CRP, CKD and Radscore. On the point scale axis, each variable was assigned a score. The overall score was calculated by adding each score. We were able to determine the probability of critical disease using the whole‐point scale.

**FIGURE 5:**
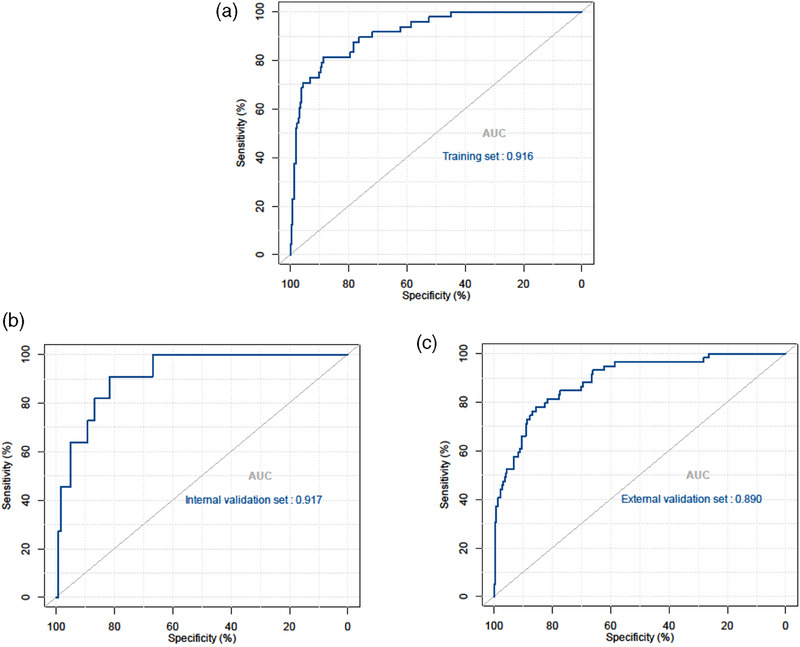
The receiver operating characteristic (ROC) curves of the nomogram in the training set (a), internal validation set (b) and external validation set (c).

The early warning nomogram was constructed based on the clinical model and radiomics model. Six independent predictors (including ALac, WBC, lymphocytes, CRP, CKD and Radscore) were incorporated into the nomogram (Figure 4). The prediction performance was quantified as AUC for 3 models, and is summarized in Table 2. The ROC curve was employed to assess the predictive ability of the constructed nomogram, which is shown in Figure 5. The result demonstrated that the AUC was 0.916 (95% CI, 0.879–0.944) in the training set, with an accuracy of 87%, sensitivity of 81% and specificity of 89%. In all sets, the nomogram model consistently demonstrated the highest performance for predicting critical disease in middle‐aged and elderly COVID‐19 patients (*p* all <0.05, DeLong test). Although the radiomics model showed the higher AUCs than clinical model in these predictions, Delong test revealed that there was no significant differences between the AUCs of these two models in the training set (*p* = 0.461, DeLong test), internal validation set (*p* = 0.889, DeLong test) or external validation set (*p* = 0.488, DeLong test).

### 3.4 Validation and evaluation of the radiomics nomogram

**FIGURE 6:**
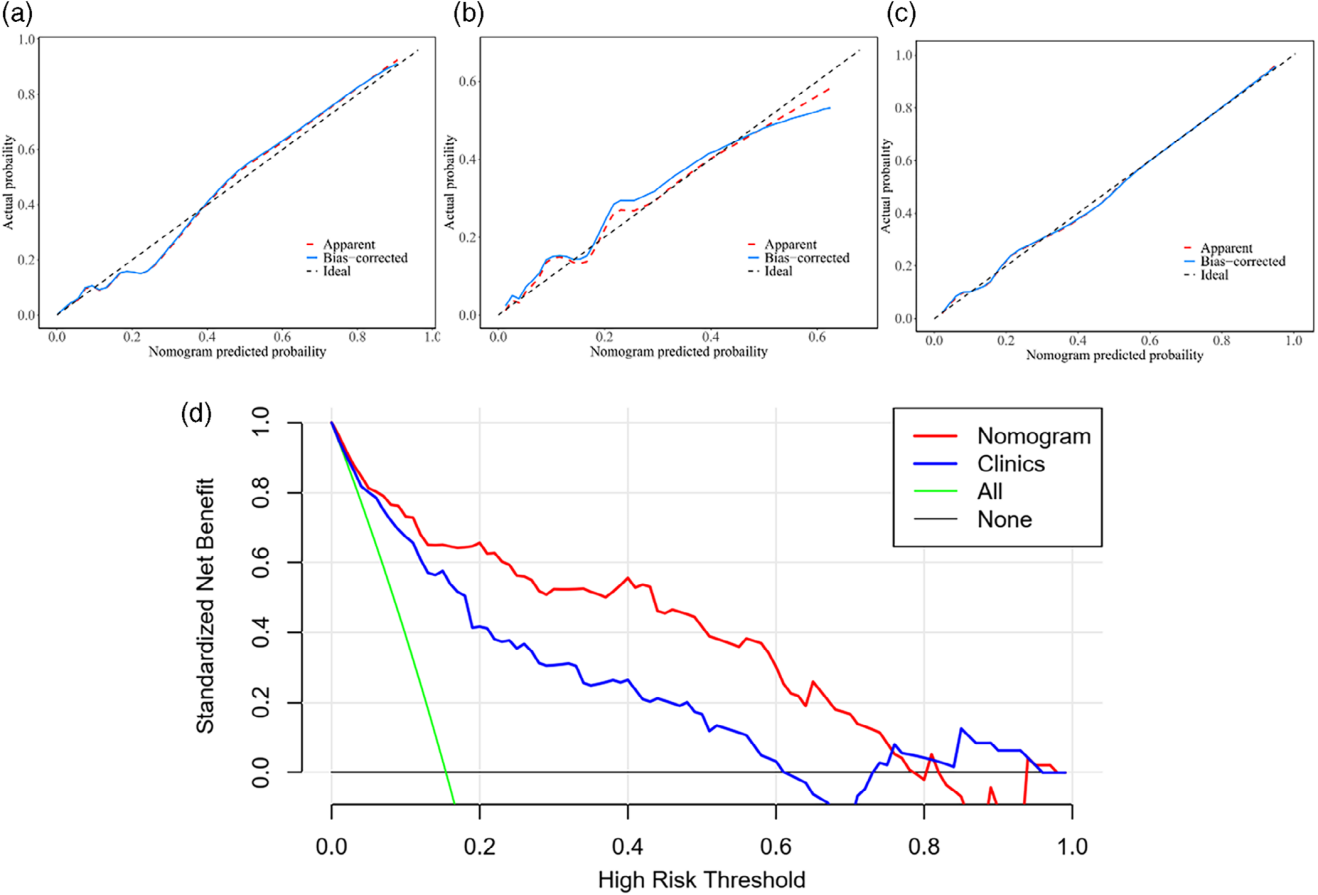
Calibration curves of the integrated nomogram in the training set (a), internal validation set (b), and external validation set (c). (d) DCA results for the integrated nomogram model and clinical model.

**FIGURE 7:**
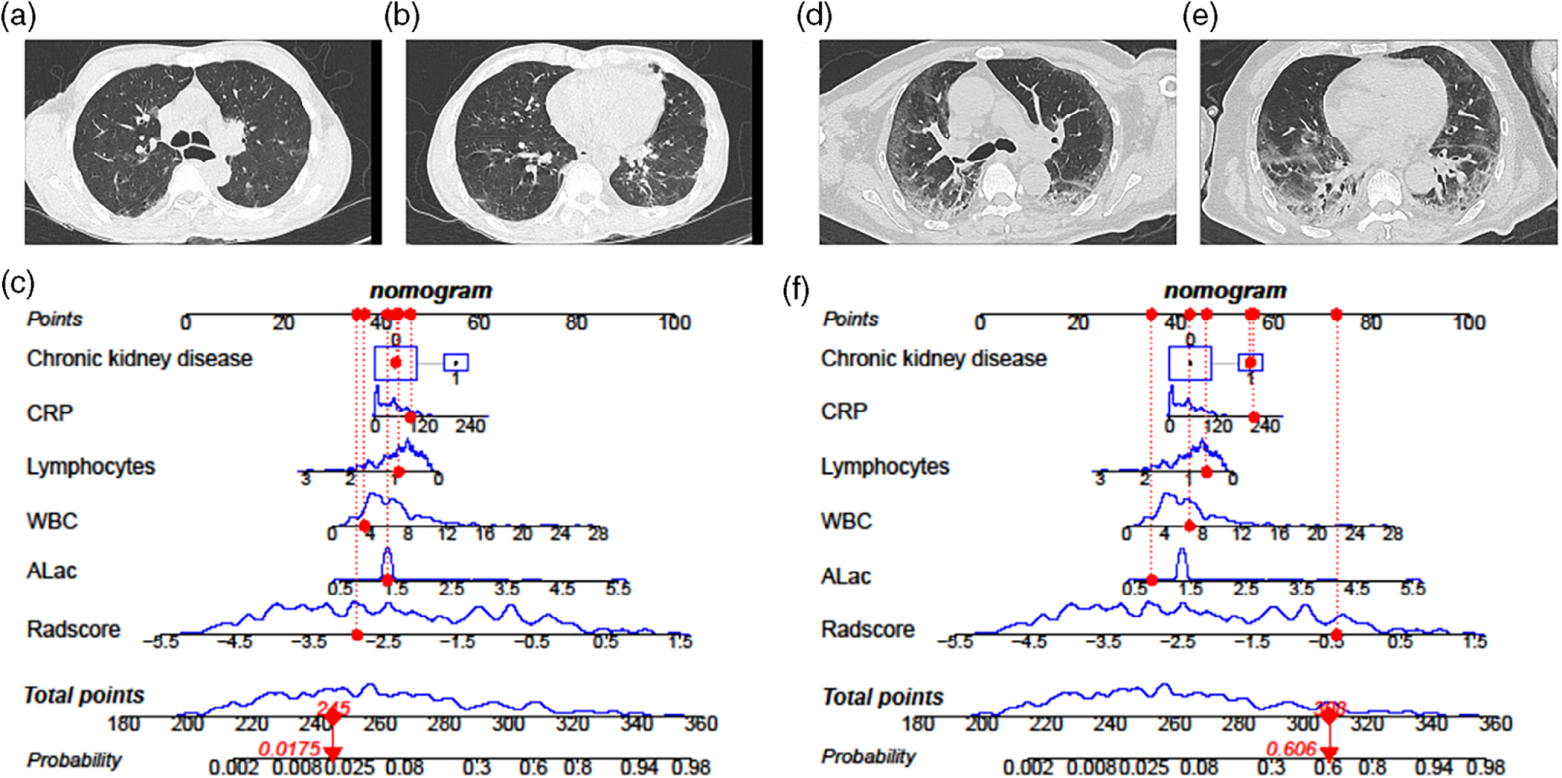
The dynamic nomogram was applied to two examples. (a–c) A 63‐year‐old non‐critical male patient, the dynamic nomogram shows the total points were 245, and the corresponding prediction probability of progressing to critical disease was 0.0175. (d–f) A 70‐year‐old critical male patient, the dynamic nomogram shows the total points were 308, and the corresponding prediction probability of progressing to critical disease was 0.606.

Internal validation and external validation of the nomogram model both demonstrated good predictive performance. The radiomics nomogram model obtained AUCs of 0.917 (95% CI, 0.857–0.958) and 0.890 (95% CI, 0.852–0.921) in the internal validation and external validation sets, respectively. Through the Hosmer‐Lemeshow test, calibration curves indicated that there was no significant departure between prediction and observation in three sets (Figure 6). Decision curve analysis (DCA) shows radiomics nomogram has better positive net benefits at threshold probabilities, which demonstrates the clinical utility of the nomogram. Two examples of applying dynamic nomogram are shown in Figure 7.

## 4 DISCUSSION

High risk early warning for middle‐aged and elderly COVID‐19 patients remains an important and topical clinical question, because it is crucial for prompt clinical intervention, thereby improving overall patient outcomes and healthcare efficiency. After feature selection of radiomic features using mRMR and LASSO, and subsequent logistic analysis of clinical factors, we found seven radiomic features and five clinical indicators that were significantly related to the critical COVID‐19 pneumonia. A CT‐based whole lung radiomics nomogram model was constructed to noninvasively predict critical COVID‐19 patients, with areas under the receiver operating characteristic curve of 0.916, 0.917, and 0.890 in the training set and the internal and external validation sets, respectively. The subtle differences in AUC among the three cohorts may be due to baseline demographic differences, as they originated from different centers. Also, we found that a high baseline radiomics model score was associated with critical pneumonia (odds ratio [OR], 2.94; 95% CI: 2.01–4.31; *p* < 0.001), indicating its potential clinical application.

In this study, age, gender, smoking status, six underlying diseases and five laboratory parameters were included. Multivariate regression analysis of clinical characteristics indicated that arterial lactate, WBC, lymphocytes, CRP and chronic kidney disease were independent predictors. Arterial blood lactate value has been regarded as one of the most important biomarkers of illness severity in patients with COVID‐19.^17^, ^18^ As expected, critical COVID‐19 patients tend to display higher lactate values than those with non‐critical. Terpos E et al.^19^ reported that lymphopenia was more common in the nonsurvivors and severe patients. Lymphopenia observed in individuals afflicted with COVID‐19 implies a dysregulated immune response.^20^, ^21^ Therefore, Lymphocytes play an important role in the pathophysiological changes of severe cases. In addition, CRP, as an acute‐phase reactive protein, reflects a hyperimmune inflammatory state, as well as WBC.^10^, ^21^ Several studies have proposed that elderly patients with chronic diseases are more likely to develop severe COVID‐19 and even die.^1^, ^2^, ^22^, ^23^ Annika et al.^24^ reported that CKD, especially end‐stage CKD, is an important risk factor for severe COVID‐19 and death after ICU admission, which is consistent with our results. Our clinical model consists of these five clinical factors. The developed clinical model performed inferior to other two models (AUC, 0.857 vs. 0.880 vs. 0.916 in training set), though the DeLong test showed that there was no significant differences between the AUCs of the clinical model and radiomics model in all three sets (*p* > 0.4, DeLong test). Notably, laboratory test results are closely related to patients’ prognosis or infection status and chronic disease represents the underlying condition of the patients. Therefore, all these clinical features are crucial for the early COVID‐19 progression assessment.

By extracting a large number of quantitative imaging features from medical images, radiomics allows for the comprehensive analysis of intra‐disease heterogeneity and enables the development of predictive models that capture complex disease phenotypes.^25^, ^26^, ^27^ Recently, radiomics has been applied to COVID‐19 pneumonia, aiding in risk stratification and treatment planning. Liang Li et al.^11^ built a radiomics model (AUC = 0.920) for the prediction of severe COVID‐19. However, they did not use laboratory test data for modeling and their nomogram was too complex to be applied clinically. Wang et al.^12^ uncovered specific radiomics features relevant to COVID‐19 pneumonia which contribute to the understanding of COVID‐19 pneumonia imaging phenotypes and found that radiomics model performed similarly to deep learning model. Gong et al.^13^ built prognosis models to predict severity outcomes and exhibited an AUC of 0.85. All these research concentrated on pneumonia segments presenting as high‐density opacities on CT images. The pathological findings of COVID‐19 pneumonia include exudative diffuse alveolar damage with alveolar and interstitial edema, alveolar fibrinous exudate with hyaline membranes, and reactive pneumocytes.^10^ Considering that SARS‐CoV‐2 may have an impact on all lung lobes and not limited to high‐density segments on CT, we use a whole‐lung radiomics analysis. Finally, our radiomics model achieved an AUC value of 0.880. Although the Delong test displayed that there was no significant difference between the clinical model and the radiomics model, from the perspective of AUC values, the radiomics model slightly outperformed the clinical model. Therefore, we have sufficient reason to think that in routine medical applications, using CT images alone for predicting the progression to critical conditions in middle‐aged and elderly COVID‐19 patients is feasible. Furthermore, compared to laboratory examinations, a single CT scan is more straightforward and practical. When integrated radiomics model and clinical model, the prediction efficiency further improved, our radiomics prediction nomogram achieved an AUC of 0.916 and an accuracy of 0.87 to identify COVID‐19 patients at high risk of progressing to critical disease, which is similar to or higher than most of the above‐mentioned studies. In particular, the DCA analysis (Figure 6d) shows that our nomogram model has a better positive net benefit than the single clinical model, demonstrating the clinical utility of the nomogram. All these results indicated that the method adopted in this study was feasible.

Our study had several limitations. First, different patients underwent different numbers of CT examinations during hospitalization, to establish a uniform baseline, our research only chose the first CT examination and ignored the dynamic changes in COVID‐19 progression. Second, when patients are admitted, they are at different stages of COVID‐19, but we only included the first laboratory examinations and CT scans after admission. Further research will stratify different time points post‐infection. Third, due to insufficient sample size, our study did not stratify patients by age, but there exists differences in risk between middle‐aged and elderly patients. Subsequent studies need to further increase sample size and focus on comparing risk differences between middle‐aged and elderly populations. Fourth, radiomics markers are constrained by their complexity and a lack of algorithmic standardization.^28^ Deep learning‐based predictive models will be developed in future research.

## 5 CONCLUSIONS

In conclusion, we built a predictive model and constructed a nomogram for predicting the occurrence of critical illness in middle‐aged and elderly COVID‐19 patients, which will contribute to personalized survival assessment and clinical management for middle‐aged and elderly patients with COVID‐19.

## AUTHOR CONTRIBUTIONS

The contributions of all authors are as follows: (I) Conception and design: Xin'ang Jiang, Jun Hu, Qinling Jiang, Li Fan, Shiyuan Liu; (II) Administrative support: Li Fan, Shiyuan Liu, Xiaoyan Xin; (III) Provision of study materials or patients: Li Fan, Shiyuan Liu, Xiaoyan Xin; (IV) Collection and assembly of data: Xinang Jiang, Yi Sun, Chao Zhou, Qianyun Ma, Jingyi Zhao, Kang Shi, Wen Yang, Xiuxiu Zhou, Yun Wang; (V) Data analysis and interpretation: Taohu Zhou, Fei Yao, Xiaoyan Xin, Li Fan; (VI) Manuscript writing: All authors; (VII) Final approval of manuscript: all authors.

## CONFLICT OF INTEREST STATEMENT

The authors have no relevant conflicts of interest to disclose.

## ETHICAL STATEMENT

This is an observational study, and individual consent was waived.

## Appendix 1

### Scanning parameters of CT equipment in two hospitals

**Table:**

CT equipment	United‐imaging	Philips		SIEMENS	GE
	uCT 760	Ingenuity CT	iCT 256	SOMATOM Force	ES
Hospital	1 or 2	2	1 or 2	1 or 2	1
Tube voltage	120 kV	120 kV	120 kV	90 kV/150Sn kV	120 kV
Tube current	AEC	AEC	AEC	AEC	AEC
Collimation	0.625 mm × 64	0.625 mm × 128	0.625 mm × 128	0.6 mm × 192	0.625 mm × 128
Rotation time	0.33s/rot	0.33s/rot	0.5s/rot	0.25s/rot	0.5s/rot
Slice thickness of Reconstruction	1/1.25 mm	1.5 mm	1/1.25 mm	1/1.25 mm	1.25 mm

Abbreviation: AEC, automatic exposure control.

## Appendix 2

### Univariable and multivariable logistic regression analysis

**Table:**

Variables	Univariate logistic analysis			Multivariate logistic analysis		
	OR	95% CI	*p*	OR	95% CI	*p*
Age	1.04	1.01–1.07	<0.001			
ALac	3.11	1.72–5.60	<0.001	2.36	1.33–4.19	0.003
WBC	1.23	1.13–1.33	<0.001	1.20	1.10–1.30	<0.001
Neutrophils	1.26	1.16–1.37	<0.001			
Lymphocytes	0.20	0.09–0.47	<0.001	0.24	0.09–0.65	0.005
CRP	1.02	1.01–1.02	<0.001	1.01	1.00–1.02	0.007
CKD	2.34	1.20–4.55	<0.001	2.43	1.11–5.35	0.027
Rad‐score	3.73	2.58– 5.38	<0.001	2.94	2.01–4.31	<0.001

Abbreviations: Alac, arterial lactate; CKD, chronic kidney disease; CRP, C‐reactive protein; WBC, white blood cell.

## Appendix 3

### 3.1

Construction of the radiomics signature. (a,b) LASSO coefficient profiles of the radiomics features. (c) The selected seven features and the absolute values of their coefficients (Figure A3).
